# Development of pachytene FISH maps for six maize chromosomes and their integration with other maize maps for insights into genome structure variation

**DOI:** 10.1007/s10577-012-9281-4

**Published:** 2012-05-16

**Authors:** Debbie M. Figueroa, Hank W. Bass

**Affiliations:** Department of Biological Science, Florida State University, Tallahassee, FL 32306-4295 USA

**Keywords:** maize, cytogenetic, fluorescence in-situ hybridization (FISH), pachytene, *Sorghum propinquum*, bacterial artificial chromosome (BAC)

## Abstract

Integrated cytogenetic pachytene fluorescence in situ hybridization (FISH) maps were developed for chromosomes 1, 3, 4, 5, 6, and 8 of maize using restriction fragment length polymorphism marker-selected *Sorghum propinquum* bacterial artificial chromosomes (BACs) for 19 core bin markers and 4 additional genetic framework loci. Using transgenomic BAC FISH mapping on maize chromosome addition lines of oats, we found that the relative locus position along the pachytene chromosome did not change as a function of total arm length, indicative of uniform axial contraction along the fibers during mid-prophase for tested loci on chromosomes 4 and 5. Additionally, we cytogenetically FISH mapped six loci from chromosome 9 onto their duplicated syntenic regions on chromosomes 1 and 6, which have varying amounts of sequence divergence, using sorghum BACs homologous to the chromosome 9 loci. We found that successful FISH mapping was possible even when the chromosome 9 selective marker had no counterpart in the syntenic block. In total, these 29 FISH-mapped loci were used to create the most extensive pachytene FISH maps to date for these six maize chromosomes. The FISH-mapped loci were then merged into one composite karyotype for direct comparative analysis with the recombination nodule-predicted cytogenetic, genetic linkage, and genomic physical maps using the relative marker positions of the loci on all the maps. Marker colinearity was observed between all pair-wise map comparisons, although marker distribution patterns varied widely in some cases. As expected, we found that the recombination nodule-based predictions most closely resembled the cytogenetic map positions overall. Cytogenetic and linkage map comparisons agreed with previous studies showing a decrease in marker spacing in the peri-centromeric heterochromatin region on the genetic linkage maps. In fact, there was a general trend with most loci mapping closer towards the telomere on the linkage maps than on the cytogenetic maps, regardless of chromosome number or maize inbred line source, with just some of the telomeric loci exempted. Finally and somewhat surprisingly, we observed considerable variation between the relative arm positions of loci when comparing our cytogenetic FISH map to the B73 genomic physical maps, even where comparisons were to a B73-derived cytogenetic map. This variation is more evident between different chromosome arms, but less so within a given arm, ruling out any type of inbred-line dependent global features of linear deoxyribonucleic acid compared with the meiotic fiber organization. This study provides a means for analyzing the maize genome structure by producing new connections for integrating the cytogenetic, linkage, and physical maps of maize.

**Electronic supplementary material:**

The online version of this article (doi:10.1007/s10577-012-9281-4) contains supplementary material, which is available to authorized users.

## Introduction

Maize (*Zea mays L.* spp. *mays*) is a well-studied cereal crop species with extensive genetic diversity (Chandler and Brendel 2002; Fu and Dooner 2002; Buckler et al. 2009; Flint-Garcia et al. 2009a, b; Schnable et al. 2009; Springer et al. 2009). The vast efforts to characterize maize’s genome structure, function, and diversity reflect its scientific and agronomic value (Walbot 2009). Three fundamentally different kinds of maps, which are publicly available for maize research, have been used to chart the positions of genetic loci in the maize genome: the cytological, genomic physical, and genetic linkage maps (Davis et al. 1999; Lawrence et al. 2004; Schnable et al. 2009; Wei et al. 2007).

The maize maps share genetic markers whose colinearity is derived from the linear structure of the chromosomes themselves, but they differ greatly in method of production, units, and the ways they are viewed and used in genetics, genomics, and selective breeding. Integrating different map types with shared markers provides a comprehensive view of genome structure while consolidating the most useful features of the various maps as such, production and integration of maps for model species, such as maize, are an active and long-term endeavor spanning many decades.

In plants, the cytological map units differ according to the conventions used in different species (reviewed by Figueroa and Bass 2010). Among the earliest characterizations of the maize genome were those based on the cytology of the meiotic pachytene-stage chromosomes (McClintock 1929); the first such maps were based on correlations between physical exchanges between chromosomes and heritable phenotypic changes in traits of maize plants (Creighton and McClintock 1931; McClintock 1930, 1931). Maize cytological maps are based on either physical features or visualization of homologous sequence hybridization along somatic or meiotic chromosomes (reviewed by Figueroa and Bass 2010). The meiotic cytogenetic maps of maize are all based on pachytene-stage chromosomes obtained from pollen mother cells, which are abundant, relatively easily obtained, and provide greater axial resolution than somatic metaphase cytogenetic maps (Chang and Neuffer 1989; reviewed by Figueroa and Bass 2010). The locus positions for the pachytene cytogenetic maps are charted in relative arm-position units referred to as centiMcClintocks (cMC; Lawrence et al. 2006); each arm measures, by definition, from 0.0 (0 %) to 1.0 (100 %).

Genetic linkage map units, based on recombination frequencies, chart loci in centiMorgans (cM); 1 cM is equal to 1 % crossover between two loci on homologous chromosomes during meiosis. Linkage maps therefore establish the order of genes on chromosomes and the degree to which linked loci are likely to segregate during meiosis. Linkage maps of maize were first developed in the 1930s (Emerson et al. 1935) and are now the most abundant type of genome maps available. Hundreds of different linkage maps have been produced in the last century (http://maizegdb.org/map.php; Davis et al. 1999; Falque et al. 2005; Lee et al. 2002; Sharopova et al. 2002; Falque et al. 2005).

More recently, genomic physical maps have been developed by fingerprint contig mapping of overlapping clones or by sequencing and assembly of deoxyribonucleic acid (DNA) fragments (Soderlund et al. 2000, 2006; Cone et al. 2002; Messing et al. 2004; Schnable et al. 2009). The loci in these maps are charted in base-pair units. The first complete genomic physical sequence maps of maize (inbred line B73) were published by Schnable et al. (2009).

For maize genomics, yet another type of map is used that includes elements of both the meiotic and cytological linkage maps: the recombination nodule (RN) maps, first produced for maize in 2003, quantify the distribution of late recombination nodules on synaptonemal complex spreads from maize pachytene chromosomes (Anderson et al. 2003). When RNs are equated with crossovers, the RN frequency map can be directly correlated with linkage map units, allowing prediction of the cytological location of a given marker on the basis of its relative position within the linkage map, as illustrated by the Morgan2McClintock Translator (Anderson et al. 2004; Lawrence et al. 2006).

Of the different types of maps for maize, the cytogenetic maps remain the least developed, despite the long history of maize cytology. This in part due to the inherent detection limit, which rarely allows for probes as small as 2.4–3.1 kb to the reliably detected (Danilova and Birchler 2008; Wang et al. 2006; and reviewed by Figueroa and Bass 2010). Pachytene and higher-resolution (e.g., fiber fluorescence in situ hybridization (FISH)) cytogenetic maps are ideal for studying plants with large duplicated blocks, for comparative mapping among the grasses, and for resolving large-area problems in genome sequence assembly (Jackson et al. 1998; de Jong et al. 1999; Feng et al. 2002; Sasaki et al. 2002; Koo et al. 2008; Szinay et al. 2008; Tang et al. 2008; Stack et al. 2009; Visser et al. 2009). The high incidence of repeat elements and corresponding low gene density presents an inherent challenge to using maize bacterial artificial chromosomes (BACs) directly on maize chromosomes; several studies have used either low copy gene amplification and blocking of repeat sequences using Cot DNA with some success (Danilova and Birchler 2008; Lamb et al. 2007; Sadder et al. 2000; and reviewed by Figueroa and Bass 2010). To address the relative deficiency in cytogenetic map development in maize, we established a single-locus cytogenetic FISH mapping for maize (Koumbaris and Bass 2003). It allowed us to overcome the signal-detection limit and produce cytogenetic FISH maps of maize restriction fragment length polymorphism (RFLP) loci using sorghum BACs (Amarillo and Bass 2007; Figueroa et al. 2011). In our previous study, we demonstrated a 75 % success rate for identifying homologous sorghum BACs and a greater than 86 % success rate for FISH mapping maize marker-selected sorghum BACs (Amarillo and Bass 2007). Here, we report using this strategy to create pachytene cytogenetic FISH maps for loci predicted to be spaced approximately 10 μm apart (Anderson et al. 2004) on maize chromosomes 1, 3, 4, 5, 6, and 8. In addition, we expand on the utility of sorghum BACs in maize cytogenetics by demonstrating their utility for mapping duplicate regions in maize. Finally, we discuss novel observations of maize genome structure that resulted from comparing our integrated pachytene cytogenetic maps to the genetic linkage and genomic physical maps of maize.

## Materials and methods

### Plant materials

Maize chromosome addition lines of oat were obtained from Drs. Howard Rines and Ron Phillips (Kynast et al. 2001; Rines et al. 2009). They carried individual disomic maize chromosomes derived from inbred line B73 (OMAd1.36, OMAd4.40, OMAd5.60, OMAd8.05), Mo17 (OMAd6.32), or Seneca 60 (OMAd3.01). These were grown in the Mission Road Research Facility greenhouse (Florida State University, Tallahassee, FL, USA) or in Conviron growth chambers as previously described (Kynast et al. 2001).

### Chromosome spreading for FISH

Meiosis-stage florets were harvested, fixed, and stored in 70 % ethanol at −20°C as previously described (Amarillo and Bass 2007). Chromosome spreads were staged and prepared as described by Figueroa et al. (2011).

### BAC FISH mapping

Homologous *Sorghum propinquum* BACs previously identified (Figueroa et al. 2011) were prepared and direct labeled as previously described (Amarillo and Bass 2007) or labeled with amino allyl dUTP (Sigma) and then with succinimidyl ester coupling Alexa-Fluor-555 as described by Invitrogen (Amine-Reactive Probes Manual). The labeled probes were then purified with the Qiagen PCR Purification Kit. Hybridizations were carried out with a direct-labeled FISH probe cocktail as previously described (Amarillo and Bass 2007). Data collection, 3D deconvolution, and digital chromosome straightening were preformed as previously described (Koumbaris and Bass 2003).

Cytogenetic locus positions were determined essentially as previously described (Koumbaris and Bass 2003; Amarillo and Bass 2007) with slight modifications. For each locus, all BAC FISH signals on the target arm were recorded in terms of their fractional distances from the centromere, or relative arm positions (0.00–1.00), along the straightened chromosome. We then displayed the resulting data as frequency histograms in bins of various sizes (10, 11, or 20 bins) to optimize the signal-to-noise ratios. Values from the bin that produced a peak exceeding the 95th percentile were averaged to yield the final locus position, rounded to two significant digits.

### Comparative mapping using standardized map units

We used relative arm positions were used to compare the different maps of maize directly. Relative map position (RMP) units, which are the percentage distance of a locus from the centromere along a given chromosome arm, are already the default units for the cytogenetic pachytene maps (our FISH maps and the RN-based maps; Anderson et al. 2004). The linkage RMP values have already been calculated for the University of Missouri Columbia (UMC) 98 linkage map (Anderson et al. 2004; Lawrence et al. 2006). The RMP values for the B73 genomic physical maps (Maize B73 RefGen_v2) were calculated from known or estimated locus coordinates from Maize Genetics and Genomics Database (Wei et al. 2009; Schnable et al. 2009; Sen et al. 2009; http://gbrowse.maizegdb.org/cgi-bin/gbrowse/maize_v2/). The relative distances on the long and short arms of the genomic physical chromosome were calculated from the total chromosome length (Mb) and the genomic location (Mb) of each locus, both obtained from the same database (Schnable et al. 2009; Sen et al. 2009). The centromere coordinates were obtained from Wolfgruber et al. (2009) as updated on the same database. From these values, the relative arm positions were calculated as shown schematically in Fig. S1, and the resulting RMPs were tabulated and used to produce the comparative map alignments.

## Results

### Maize chromosome arm ratios in oat addition lines are consistent with those previously reported for maize

Previous work with the disomic maize addition lines of oat (OMAd lines) showed that the arm ratio of maize chromosome 9 in the oat background is comparable to that typically seen with maize chromosomes obtained directly from maize plants (Koumbaris and Bass 2003; Amarillo and Bass 2007), but the arm ratios for the targets of this study (chromosomes 1, 3, 4, 5, 6, and 8) in the oat background have not been previously described. We therefore first examined the arm ratios for these six maize chromosomes and found that they were in general agreement with those from previously published studies, as shown in Table S1. The arm ratio of OMAd chromosome 4 appeared to be smaller than that observed in previous studies, whereas chromosomes 1 and 5 had slightly higher arm ratios. The minor discrepancies observed may be experimental artifacts, as evidenced by the different reported arm ratios between studies using the same maize inbred line, KYS (Rhoades 1950; Chen et al. 2000; Sadder and Weber 2001; Wang et al. 2006). The greatest variation in arm ratio is seen for chromosome 6 (Table S1) and is probably due to the presence of the nucleolus organizer region and other variable chromomeres in the short arm of this chromosome (Phillips et al. 1974). Aside from chromosome 6S, which was omitted from this study, we concluded that our OMAd material was suitable for pachytene FISH mapping and consistent with previous studies.

### Cytogenetic FISH mapping procedure using maize addition lines of oat

**Fig. 1:**
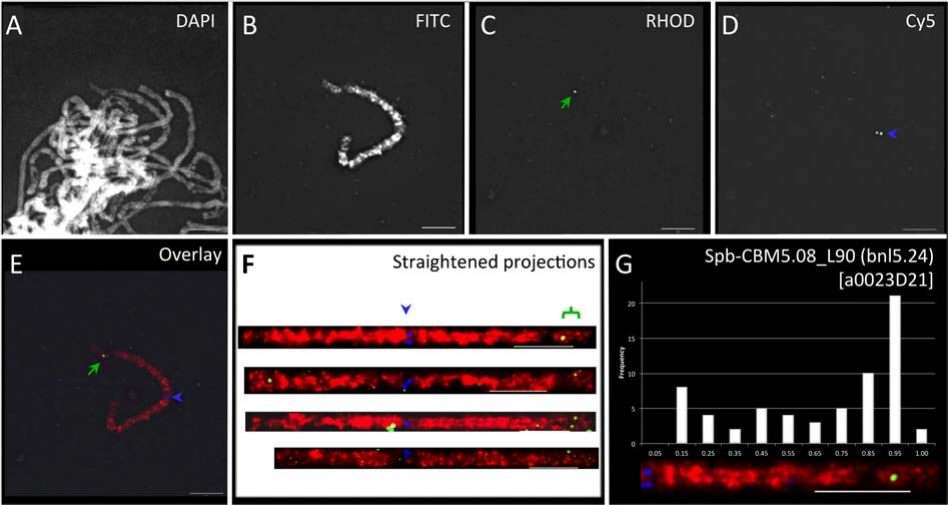
FISH mapping of maize CBM5.08 with sorghum BAC a0023D21. **a** A DAPI-stained image of spread pachytene chromosomes from OMAd5.60. **b** FITC image showing maize chromosome 5 direct labeled with Alexa-488-dUTP-KWF total maize DNA. **c** Rhodamine image from A455-labeled sorghum BAC FISH signals (*green arrows*). **d** Cy-5 image of centromere FISH signal (*blue arrowhead*) with direct-labeled CentC. **e** Three-color overlay of the (*red*), rhodamine (*green*), and Cy-5 (*blue*) images. **f** Straightened projection of the maize chromosome from (**e**) along with three other representative images and the locations of the centromere (*blue arrowhead*) and CBM5.08 BAC FISH signals (*green bracket*) are indicated. **g** The representative chromosome arm with BAC signal, histogram of all signals observed on the 5S arms measured, along with the resulting cytogenetic locus name (in *brackets*) are shown. All *scale bars* represent 5 μm

Maize-marker-selected sorghum BACs were direct labeled for use as FISH probes and hybridized, as part of a three-probe cocktail, to pachytene spreads from OMAd lines. Figure 1 illustrates our procedure of using *S. propinquum* BAC (a0023D21) FISH of the CBM5.08 locus on a maize B73 chromosome-5 addition line of oat (OMAd5.60^B73^). The three-probe cocktail included total DNA-painting probe from the knobless Wilbur’s Flint line of maize (Fig. 1b, FITC image), the centromere-specific probe centC (Fig. 1d, Cy-5 image; Ananiev et al. 1998), and the target maize marker-selected homologous sorghum BAC probe (Fig. 1c, Rhodamine image). The nuclei were counterstained with DAPI (Fig. 1a, DAPI image), and 3D deconvolution images were collected as previously described (Koumbaris and Bass 2003; Amarillo and Bass 2007). A three-color overlay of the maize chromosome GISH (red), CentC FISH (blue), and BAC FISH signal (green) is shown (Fig. 1e). Images of unobstructed pachytene chromosomes were then manually traced within the 3D data set, and the path of the fiber was computationally straightened (Fig. 1f). The cytogenetic positions of the BAC signals were measured on the straightened fiber images. The resulting values were converted to histograms, and the regions with signals above background were averaged to yield the mapping location (Fig. 1g). In this example, the maize CBM5.08 locus was indirectly FISH mapped, with a sorghum BAC (a0023D21), to 90 % of the distance along the long arm. The cytogenetic position for this locus is denoted spb-CBM5.08_L90 (bnl5.24) according to sorghum BAC FISH nomenclature first described by Koumbaris and Bass (2003). This location is in general agreement with the value of 5L.91 predicted from RN frequency mapping in maize (Anderson et al. 2004).

### Chromosome axial contraction is uniform at the resolution of pachytene FISH

**Fig. 2:**
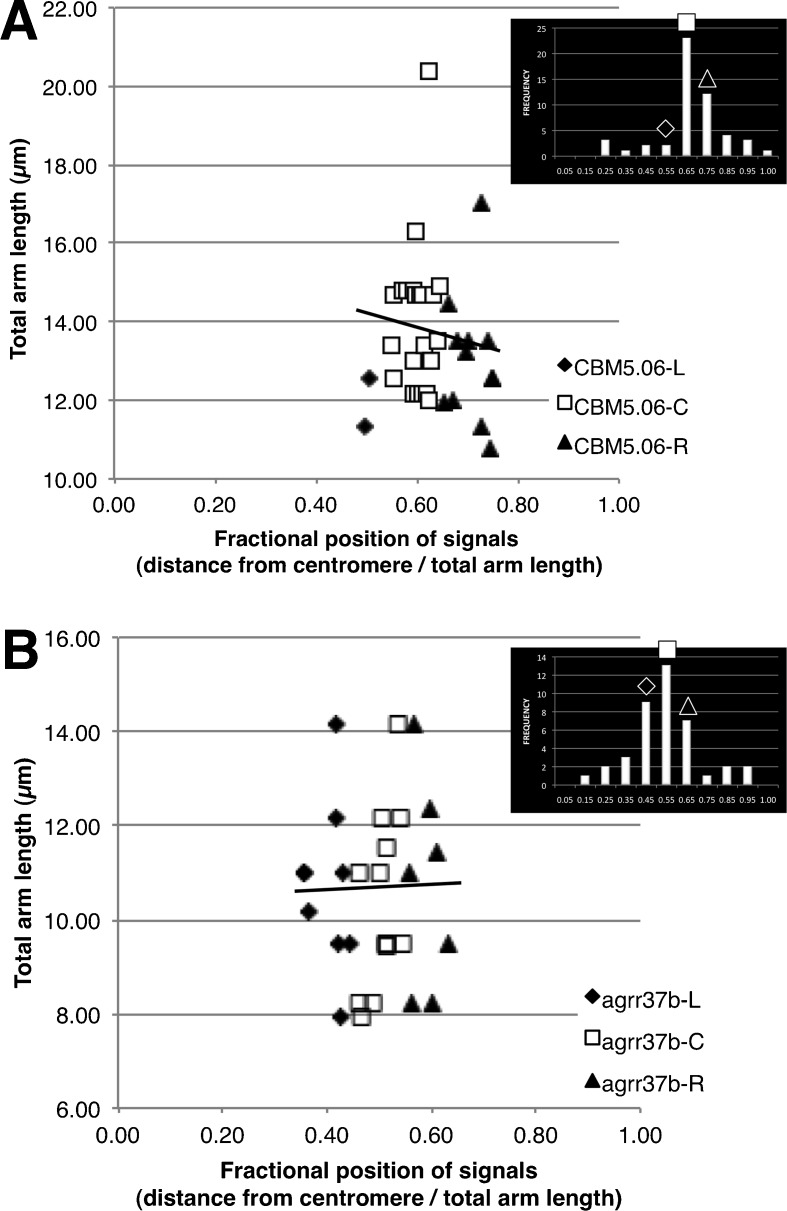
The fractional positions of the all the signals from the bin used to compute the cytogenetic FISH locus for signals in the center (*C*, *squares*) as well as from the bins to the left (*L*, *diamonds*) and right (*R*, *triangles*) plotted against the total length. **a** A case (*CBM5.06*) where one bin adjoining that used for cytogenetic mapping was markedly raised (a one-shoulder case). **b** A case (*CBM4.05*) where both adjoining bins were raised (a two-shoulder case). The linear regression *line* for the points in all of the bins is also shown

We then asked whether the relative position of a FISH mapped locus remains constant, even on chromosomes of different absolute lengths. It is conceivable that the variation in length of a given chromosome arm may be the result of uneven axial contraction along the pachytene chromosome fiber. If so, then the map position would not be fixed but would instead vary with chromosome-arm length throughout early and late pachytene of meiosis. To determine whether this is the case, we examined the population of measurements for two loci by plotting chromosome-arm length against cMC position. The loci examined were CBM5.06, which represents a FISH-mapped locus where the one of the adjoining bins has a concurrent increase in signal number relative to the other bins along the chromosome, referred to as having one shoulder, and CBM4.05, which had two shoulders, as shown in Fig. 2. Linear regressions of the FISH signals on absolute arm lengths, including those in the adjoining left and right bins, revealed that for CBM5.06, arm length was slightly but not significantly negatively correlated with relative signal position (slope of −5.4, *R* ^2^ = 0.034, *p* = 0.28; Fig. 2a). Analysis of the bin used to map CBM4.05, along with the two shoulders, also failed to find a correlation between arm length and relative signal position (slope of 1.4, *R* ^2^ = 0.0028, *p* = 0.79; Fig. 2b). In both cases, the lack of correlation suggests that no obvious relationship exists between the relative arm position of a locus and the total arm length, consistent with the idea of uniform axial contraction of pachytene chromosome fibers at this scale. Therefore, the cytogenetic map positions of these test loci on chromosome arms 5L and 4L, and presumably on other chromosome arms, are not sensitive to changes in the total arm length throughout pachytene stage of meiosis.

### Cytogenetic FISH mapping of six maize chromosomes

**Fig. 3:**
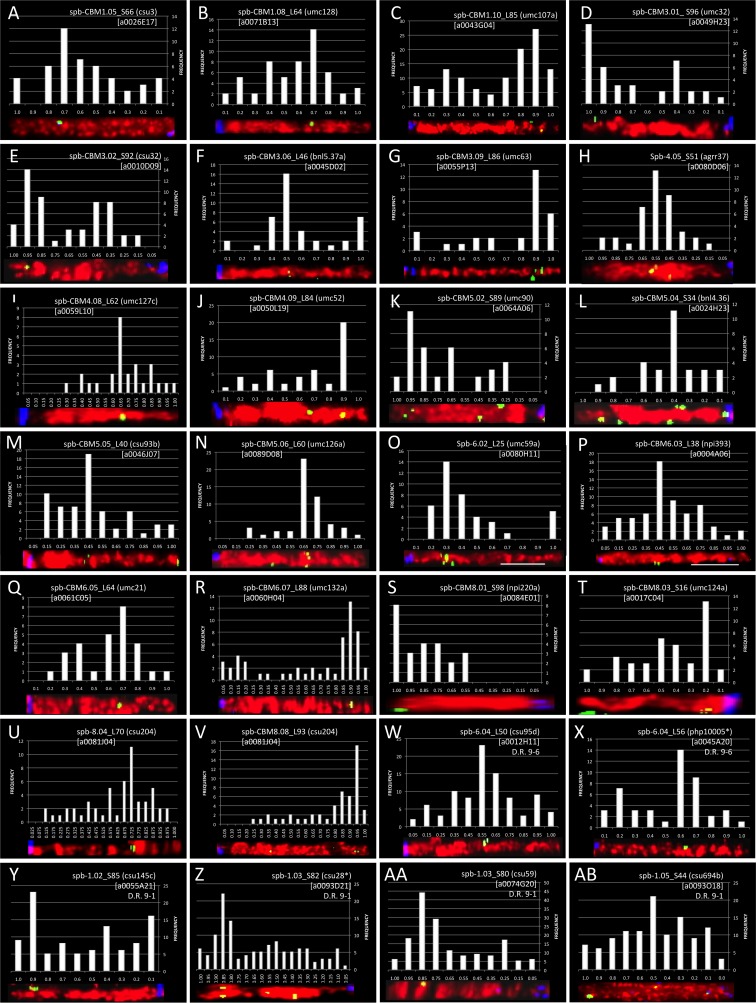
Histograms, locus names, and representative chromosome images for the FISH-mapped loci. Each panel displays the histogram used to delimit the FISH loci used to tabulate the mapping location, a straightened projection of a representative chromosome arm, and the resulting locus name. Images are organized according to chromosome and arm on which loci reside: chromosomes 1 (*A–C*), 3 (*D–G*), 4, (*H–J*), 5 (*K–N*), 6 (*O–R*), and 8 (*S–V*). Synteny-mapped loci are also shown for chromosomes 1 (*W–Z*) and 6 (*AA*, *AB*). *Asterisks* indicate FISH mapping of sorghum BACs in regions where the duplicate locus has been lost

Image collection, chromosome straightening, signal tabulation, and histogram development were performed for the 23 framework loci that were FISH mapped. Figure 3 shows the resulting signal histograms, cytogenetic locus names, and representative chromosome images for the FISH-mapped loci (A–V). We plotted all signals in 10 or 20 equal-sized bins to maximize the resolution and signal-to-noise ratio when identifying the FISH mapping location. Occasionally, FISH signals from some loci clustered near the boundaries of two adjacent bins, causing the bins to peak concurrently. To map these loci, we phase-shifted the bins by 0.05, thus producing a plot with 11 bins where the first and last bins were half the size of the remaining nine bins, to permit complete capture of the FISH signals in one bin. For most of the FISH-mapped loci, ten bins produced a peak that exceeded a 95 % confidence interval and were used to identify the locus FISH mapping position (Fig. 3(A–D, F–G, J, L, O, Q, T)); 11 bins were necessary for mapping seven loci (Fig. 3(E, H, K, M–N, P, S)). In addition, we increased the FISH-mapping resolution for four loci by using 20 bins (Fig. 3(I, R, U–V)). Table 1 lists all of the core bin markers (CBMs) and within bin markers that were FISH mapped.

**Table 1:** Loci mapped by fluorescence in situ hybridization (FISH) during the present study

Bin^a^	Locus	Probe name^b^	FISH probe selected^c^	FISH locus (cMC (*n*))^d^	Cytolocus name
CBM1.05	csu3	p-csu3	a0026E17	1S.66 (12)	spb-CBM1.05_S66 (csu3)
CBM1.08	umc128	p-umc128	a0071B13	1L.64 (14)	spb-CBM1.08_L64 (umc128)
CBM1.10	umc107a	p-umc107	a0043G04	1L.85 (27)	spb-CBM1.10_L85 (umc107a)
CBM3.01	umc32a	p-umc32	a0049H23	3S.96 (13)	spb-CBM3.01_S96 (umc32a)
CBM3.02	csu32a	p-csu32	a0010D09	3S.92 (14)	spb-CBM3.02_S92 (csu32a)
CBM3.06	bnl5.37a	p-bnl5.37	a0045D02	3L.46 (16)	spb-CBM3.06_L46 (bnl5.37a)
CBM3.09	umc63a	p-umc63	a0055P13	3L.86 (13)	spb-CBM3.09_L86 (umc63a)
4.05	agrr37b	p-agrr37	a0080D06	4S.51 (6)	spb-4.05_S51 (agrr37b)
CBM4.08	umc127c	p-umc127	a0059L11	4L.62 (8)	spb-CBM4.08_L62 (umc127c)
CBM4.09	umc52a	p-umc52	a0050L19	4L.84 (20)	spb-CBM4.09_L84 (umc52a)
CBM5.02	umc90	p-umc90	a0064A06	5S.89 (20)	spb-CBM5.02_S89 (umc90)
CBM5.04	bnl4.36	p-bnl4.36	a0024H23	5S.35 (11)	spb-CBM5.04_S35 (bnl4.36)
5.05	csu93b	p-csu93	a0046J07	5L.40 (19)	spb-5.05_L40 (csu93b)
CBM5.06	umc126	p-umc126	a0089D08	5L.60 (23)	spb-CBM5.06_L60 (umc126a)
CBM5.08	bnl524a	p-bnl5.24a	a0023D21	5L.90 (21)	spb-CBM5.08_L90 (bnl5.24a)
6.02	umc59a	p-umc59	a0080H11	6L.25 (14)	spb-6.02_L25 (umc59a)
CBM6.03	npi393	p-G23A-06	a0004A06	6L.38 (13)	spb-CBM6.03_L38 (npi393)
CBM6.05	umc21	p-umc21	a0061C05	6L.64 (8)	spb-CBM6.05_L64 (umc21)
CBM6.07	umc132a (chk)	p-umc132	a0060H04	6L.88 (13)	spb-CBM6.07_L88 [umc132a (chk)]
CBM8.01	npi220a	p-G10F-01	a0084E01	8S.98 (8)	spb-CBM8.01_S98 (npi220a)
CBM8.03	umc124a (chk)	p-umc124	a0017C04	8S.16 (13)	spb-CBM8.03_S16 [umc124a (chk)]
8.04^e^	csu204 (uce)	p-csu204	a0081J04	8L.70 (11)	spb-8.04_L70 [csu204 (uce)]
CBM8.08	npi414a	p-npi414	a0073D02	8L.93 (17)	spb-CBM8.08_L93 (npi414)

^a^Genetic bin housing the locus; Core Bin Markers on the B73 RefGen_v2 pseudomolecule are denoted “CBM” (http://www.maizegdb.org/cgi-bin/bin_viewer.cgi); these are used to define genetic bins as originally defined by Gardiner et al. (1993)

^b^Maize restriction-fragment length polymorphism used to identify homologous sorghum BACs by hybridization to the YRL filter set (Lin et al. 1999).

^c^Sorghum BAC selected from YRL filter screens performed by Figueroa et al. (2011)

^d^The FISH centiMcClintock (cMC) locus is identified by three or more alphanumeric characters written as *xy*.*z*, where *x* is the chromosome number, *y* is the S or L (short or long chromosome arm), and *z* is the mean fractional distance of the signals along the chromosome arm used to determine the locus position. *n* the number of FISH signals used to calculate the mean fractional distance

^e^csu204 was selected for FISH mapping of the large region between CBM8.03 and CBM8.08 on the long arm of chromosome 8, and YRL filter hybridization resulted in the detection of the following contigs (BACs): 645 (a0081J04 and a0012I24), 816 (a0028G14, a0089P13, and a0045P02), and 59 (a0086P24 and a0063K20)

We attempted to map loci predicted to be spaced approximately 10 μm apart on the maize pachytene chromosome (Anderson et al. 2004), but a few of the selected maize RFLPs failed to identify homologous sorghum BACs with the Southern hybridization technique (Figueroa et al. 2011). The regions between the centromere and CBM1.08 on the long arm of chromosome 1 and those between the centromere and CBM8.08 on the long arm of chromosome 8 are examples. For these cases, nearby RFLP markers (csu542 and csu204) were chosen to identify homologous sorghum BACs that could be used to bridge the approximately 15-μm gaps on the long arms of chromosomes 1 and 8, respectively. Of these, the csu542 maize marker did not yield a clear homologous sorghum BAC Southern hybridization pattern, but the csu204-selected sorghum BAC was successfully used as an alternate, mapping to 8 L.70 (Fig. 3(U); Table 1).

### Sorghum BAC FISH for mapping variably conserved duplicate regions in maize

The maize genome is known for its extensive large-scale segmented duplications (Helentjaris et al. 1988). Within these duplicated segments, the degree of conserved gene order, synteny, can vary considerably (Gaut 2001). This feature of the maize genome raises an interesting possibility for our mapping technique; one sorghum BAC may hybridize to and be sufficient for FISH mapping of two different loci in two different chromosome-addition lines of maize. We tested this idea using sorghum BACs that were previously used as FISH probes on chromosome 9 (Amarillo and Bass 2007). Chromosome 9 shares syntenic duplicate segments with chromosomes 6 and 1. Of the 32 mapped BACs on chromosome 9, we identified seven that fall within a large syntenic block between chromosomes 9 and 6, here designated “duplicate region 9–6,” as shown in Fig. S2 and listed in Table S2. Similarly, we identified 17 BACs that fall within a large syntenic block between chromosomes 9 and 1, here designated “duplicate region 9–1” (Fig. S2; Table S2).

These two large syntenic blocks, along with two additional smaller ones, have been annotated with known or estimated CBM locations (Fig. S2a). Of the two large syntenic blocks, duplicate region 9–6 exhibits rather low shared-marker density, as demonstrated by the few connecting lines between the regions (Fig. S2b), whereas duplicate region 9–1 exhibits extensive synteny and a large proportion of shared markers (Fig. S2c). We expected that the latter would be better suited to mapping with BACs selected for chromosome 9. In addition, within each of these blocks are cases in which the genetic marker on chromosome 9, used to select the sorghum BAC, did not have a corresponding duplicate locus on the physical map of chromosome 6 or 1. We were therefore able to examine four different intraspecies BAC FISH mapping scenarios, listed here in order from greatest to least likelihood of success, in principle. These four scenarios are represented by cases where the sorghum BACs were selected by maize RFLP markers (1) with a known duplicate in a dense syntenic block (csu94, csu59, and csu145), (2) without a known duplicate in a dense syntenic block (csu28), (3) with a known duplicate in a sparse syntenic block (csu95), and (4) without a known duplicate within a sparse syntenic block (php10005).

Surprisingly, all six BACs selected were successfully used for synteny mapping as shown in Table 2 and Fig. 3(W–AB). Interestingly, csu694b, known to be present in the syntenic segment 9–1, appeared to have slightly higher than normal background levels (Fig. 3(AB)), whereas the BACs from markers lacking a known duplicate in the syntenic region (Fig. 3(X, Z)) produced histograms with some of the lowest background levels. Even the BAC in the sparse syntenic region whose selective marker (php10005) had no known duplicate on chromosome 6 was successfully FISH mapped. In fact the overall signal-to-noise ratios were similar to those seen when homologous sorghum BACs are mapped onto their original target regions (Fig. 3(A–V)). Taken together, our results demonstrate that, at least for the loci studied here, sequence conservation in the maize marker-selected sorghum BACs was sufficient for FISH mapping of duplicated syntenic regions of maize, even when the maize marker used to select the BAC was not duplicated on the syntenic segment.

**Table 2:** Syntenic loci FISH mapped in the present study

Maize 9 bin	Maize 9 locus	Duplicate bin	Duplicate locus name	Synteny block with 9^a^	Sorghum BAC FISH probe^b^	FISH locus (cMC (*n*))^c^	Cytolocus name
9.01	php10005	6.04	(no mapped duplicate)	Sparse	a0045A20	6L.56 (14)	spb-6.04_L56 (php10005^d^)
9.01	csu95	6.04	csu95d	Sparse	a0012H11	6L.50 (23)	spb-6.04_L50 (csu95d)
9.04	csu694 (uce)	1.05	csu694b(uce)	Dense	a0093O18	1S.44 (21)	spb-1.05_S44 (csu694b)
9.06	csu59	1.03	csu59b	Dense	a0074G20	1S.78 (44)	spb-1.03_S80 (umc59b)
9.06	csu145	1.02–1.03	csu145c(pck)	Dense	a0055A21	1S.85 (23)	spb-1.02_S85 (csu145c)
9.06	csu28 (rps22)	1.03	(no mapped duplicate)	Dense	a0093D21	1S.82 (22)	spb-1.03_S82 (csu28^d^)

^a^The density of markers known to be duplicated on chromosome 9 and the syntenic region on the duplicated chromosome

^b^Sorghum BAC used to FISH map locus on chromosome 9 by Amarillo and Bass (2007)

^c^The FISH cMC locus is identified by as described in Table 1

^d^Absence of the maize locus used to select the homologous sorghum BAC from the FISH mapped duplicate segment

**Fig. 4:**
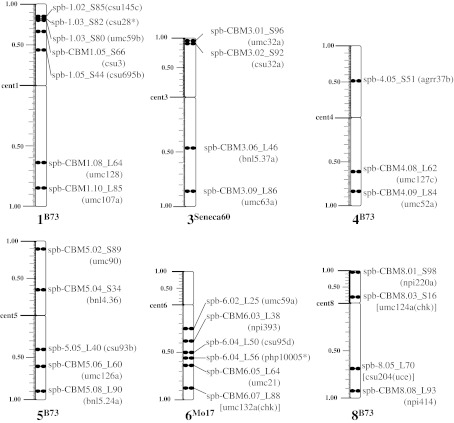
Transgenomic cytogenetic FISH maps of six maize pachytene chromosomes. Karyotypes of maize chromosomes 1, 3, 4, 5, 6, and 8 with all the loci FISH mapped are presented along with the cytogenetic names. The chromosomes are shown with their short arms on top and long arm on the bottom. The arm-ratio ruler (in cMC) is displayed along the left side of every chromosome arm; the chromosome end is designated 1.00. The *asterisk* indicates that the maize locus used to select the homologous sorghum BAC was not present on the FISH-mapped duplicate segment

A summary cytogenetic FISH map karyotype is presented in Fig. 4 for all of the loci FISH mapped on the six maize chromosomes studied. The karyotypes reflect the proportional arm ratios as well as the fractional distance of each FISH-mapped locus along the chromosomes. A total of 29 loci were FISH mapped onto chromosomes 1, 3, 4, 5, 6, and 8, three to seven per chromosome at a spacing of approximately 10 μm, providing the first pachytene FISH maps of multiple genetic markers for these chromosomes.

### Integration and analysis of marker positions across multiple maize maps

**Fig. 5:**
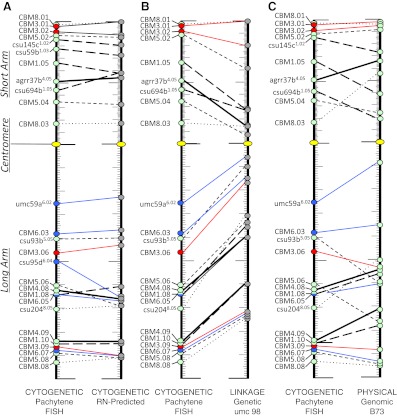
Comparison of the pachytene FISH composite karyotype to various maize maps in standardized RMP units. The pachytene FISH composite karyotype is shown first in each panel, and the loci are connected by lines to their RN-predicted RMPs (**a**), their RMPs on the UMC 98 linkage maps (**b**), or their RMPs on the B73 genomic physical maps. The karyotypes are displayed as short arm-centromere-long arm with the centromere indicated by a *yellow oval*. The maize inbred *line* used in pachytene FISH for each locus is denoted by colored *circles*: B73 (*green*), Seneca 60 (*red*), and Mo17 (*blue*). *Lines* connecting loci on two maps differentiate between the different B73-derived chromosomes 1 (*long-dashed*), 4 (*thick*), 5 (*short-dashed*), and 8 (*dotted*) as well as the Seneca 60 (chromosome 3, *red*) and Mo17 (chromosome 6, *blue*) derived chromosomes

To integrate our pachytene FISH maps and to compare them directly to other maize maps, we used a standardized map-unit system in which the fractional distance of each locus along the chromosome arm in each map is used, denoted here as RMP units. The pachytene cytogenetic map coordinates have historically been expressed in RMP units, fractional distance along an arm, more recently termed cMC by Lawrence et al. (2006). The loci that we pachytene FISH mapped and their corresponding RMP values on several other maps are summarized in Table 3. Figure 5 shows composite karyotype alignments between the pachytene FISH maps and each of the other maps: the RN-predicted cytogenetic map (Fig. 5a), the UMC 98 linkage map (Fig. 5b), and the B73 genomic physical map (Fig. 5c). Loci from different chromosomes are all anchored to a single short- and long-arm scaffold.

**Table 3:** Comparison of cytogenetic RMPs to RN-predicted, linkage, and physical RMPs

Bin	Locus	Arm^a^	Relative map positions (%)				Deviation from pachytene FISH RMP^b^		
			Cytogenetic FISH^c^	Cytogenetic RN-predicted^d^	Linkage UMC98^e^	Genomic physical^f^	Cytogenetic RN-predicted	Linkage UMC98	Genomic physical
CBM1.05	csu3	S	66^B73^	58	28	39	8	38	27
CBM1.08	umc128a	L	64^B73^	62	36	56	2	28	8
CBM1.10	umc107a (croc)	L	85^B73^	85	60	78	0	25	7
CBM3.01	umc032a	S	96^Seneca 60^	100	100	98	−4	−4	−2
CBM3.02	csu32a	S	92^Seneca 60^	89	80	96	3	12	−4
CBM3.06	bnl5.37a	L	46^Seneca 60^	43	17	53	3	29	−7
CBM3.09	umc63a	L	86^Seneca 60^	90	72	88	−4	14	−2
4.05	agrr37b	S	51^B73^	54	15	66	−3	36	−15
CBM4.08	umc127c	L	62^B73^	66	40	54	−4	22	8
CBM4.09	umc52a	L	84^B73^	84	60	71	0	24	13
CBM5.02	umc90	S	89^B73^	87	61	92	2	28	−3
CBM5.04	bnl4.36	S	35^B73^	32	7	24	2	27	10
5.05	csu93b	L	40^B73^	40	10	59	0	30	−19
CBM5.06	umc126	L	60^B73^	69	34	76	−9	26	−16
CBM5.08	bnl524a	L	90^B73^	91	74	94	−1	16	−4
6.02	umc59a	L	25^Mo17^	23	6	9	2	19	16
CBM6.03	npi393	L	38^M017^	37	15	35	1	23	3
CBM6.05	umc21	L	64^Mo17^	67	40	60	−3	24	4
CBM6.07	umc132a (chk)	L	88^Mo17^	90	73	93	−2	15	−5
CBM8.01	npi220a	S	98^B73^	100	100	96	−2	−2	2
CBM8.03	umc124a (chk)	S	16^B73^	17	14	56	−1	2	−40
8.04	csu204 (uce)	L	70^B73^	69e	31	54	1	39	16
CBM8.08	npi414a	L	93^B73^	91	75	95	2	18	−2
Duplicate bin									
6.04	csu95d	L	50^Mo17^	64.5–65.4	–	–	−15		
1.05	csu694b (uce)	S	44^B73^	58	27	19	−14	17	25
1.03	csu59b	S	78^B73^	71–73	–	–	6		
1.02	csu145c (pck)	S	85^B73^	80–81	–	74	5		11

^a^Chromosome arm on which locus resides, long (L) or short (S)

^b^(FISH RMP) − (comparative map RMP)

^c^cMc, as RMPs with the maize inbred line chromosome source in OMA line indicated in superscript

^d^By Anderson et al. (2004) except for markers csu95d (pl1 and umc248), csu59b (P1 and umc66), and csu145c (csu315c and umc11a), whose values were predicted by means of the Morgan2McClintock Translator with closest flanking markers on the umc98 map

^e^RMP calculated from Anderson et al. (2003) RN data as published by the Morgan2McClintock Translator with umc98 linkage map cM values, Maize Version 2.0 (v1.0; Lawrence et al. 2006)

^f^Physical RMPs from Supplemental Table 2 determined as described in Supplemental Fig. 1

In comparing the composite pachytene FISH karyotype to the RN-based cytogenetic map (Anderson et al. 2004), we found that the RMPs were remarkably similar, showing comparable distributions along the chromosome arms (Fig. 5a; Table 3). The same was true for multiple genotypes and across several chromosomes, consistent with previous observations for chromosome 9 (Anderson et al. 2004). For example, CBMs 1.10, 4.09, and 5.05 all had the same RMP on the two maps. In fact, none of the pachytene FISH comparisons to the RN-based predictions revealed differences of more than 16 RMP units; most were less than 10 RMP units. Of the CBM markers, CBM1.05^B73^ and CBM5.06^B73^ exhibited the greatest difference between the maps. Interestingly, the two markers that differed most widely were those from the BAC synteny mapping experiment (csu95d^Mo17^ and csu694b(uce)^B73^). Finally, the linear orders of all of the loci along a given chromosome arm were the same in our pachytene FISH maps and the RN-predicted cytogenetic maps.

We next compared the distributions of shared markers on a widely used maize linkage map, UMC 98, to our cytogenetic maps (Table 3; Fig. 5b). We found that centromere-linked loci mapped to more distal RMPs in the cytogenetic maps than in the linkage maps. This pattern of reduced recombination around centromeres was expected on the basis of prior studies in maize and other plant species (Davis et al. 1994; Gill et al. 1996a, b; Copenhaver et al. 1999; Künzel et al. 2000; Cheng et al. 2001; Tenaillon et al. 2002; Anderson et al. 2003; Wu et al. 2003; Khrustaleva et al. 2005). Only the two loci nearest to the telomere (Fig. S2B, CBM3.01^Seneca60^ and CBM8.01^B73^) were exceptions to this trend.

Finally, we compared the distributions of shared markers on the B73 genomic physical map and our cytogenetic maps (Table 3; Fig. 5c). For this comparison, we converted the genomic coordinates into RMPs as illustrated in Fig. S1 and summarized in Table S3. As expected, the comparisons revealed congruent ordering but with considerable variation in spacing, even though both map types are physically based. The maps showed the best RMP agreement for loci near the telomeres, especially with chromosomes 3^Seneca60^, 5^B73^, 6^Mo17^, and 8^B73^. Of the arms considered, the loci on 6L (6^Mo17^, Fig. 5c) showed the best agreement (differing by less than 5 RMP units) between markers on these maps; the one exception, UMC 98a^6.02^, showed a difference of 16 RMP units.

Interestingly, we observed regions where loci mapped closer to the centromere on the cytogenetic maps than on the RMP-based physical maps. Examples include bin4.05, CBM5.05 (5L.40), and CBM8.03, all of which were mapped to a B73 chromosome in the addition lines. This pattern was also seen for all the loci FISH mapped to chromosome 3^Seneca60^ (Fig. 5c), but the opposite pattern was observed with the loci mapped onto the B73 chromosome arms 1S, 1L, and 4L and most of the loci on the Mo17 chromosome 6.

## Discussion

A significant obstacle for the development of cytogenetic FISH maps has been the inherent detection limit of FISH, which make reliable detection of fluorescent probes smaller than a few Kb difficult (reviewed by Figueroa and Bass 2010). In this study, we continued to employ transgenomic BAC FISH to overcome this obstacle (Koumbaris and Bass 2003; Amarillo and Bass 2007; Figueroa and Bass 2010; Figueroa et al. 2011). We FISH mapped sorghum BACs onto maize chromosomes that were genetically isolated as doubled haploids in oat plants (Kynast et al. 2001; Koumbaris and Bass 2003; Rines et al. 2009). These OMAd lines provide stable propagation of the maize chromosomes as judged by retention of all tested simple sequence repeat markers (Riera-Lizarazu et al. 2000; Kynast et al. 2001) and preservation of chromosome 6 centromere organization (Jin et al. 2004). Cytogenetically, we found that the pachytene arm-length ratios of the OMAd lines we used were similar to those previously reported from various maize inbred lines (Table S1; Koumbaris and Bass 2003). These observations suggest that meiotic chromosome packaging is uniform at the resolution of pachytene FISH, even when propagated in a distally related genus.

We have produced new transgenomic cytogenetic pachytene FISH maps with approximately 10-μm spacing on six different maize chromosomes. These new maps establish empirically determined anchors between the linkage and cytological maps of maize, which are particularly valuable for the extensive collection of translocation stocks of maize, most of which have chromosome break points mapped cytologically (cMC positions) but not genetically (Coe 1994). Our mapping data therefore increases the prospects for using these defined translocation stocks in genetic, genomic, or gene-dosage studies as well as breeding efforts (Sheridan and Auger 2006)

Maize is an ancient tetraploid that has undergone a relatively recent large-scale duplication event (Gaut et al. 2000; Blanc and Wolfe 2004; Swigonova et al. 2004; Wei et al. 2007; Schnable et al. 2009, 2011). After this duplication, extensive gene loss and rearrangements are thought to have contributed to diploidization, with duplicate regions appearing to sort between relatively high-expressing genes or low-expressing genes and a concomitant relative retention rate (Schnable et al. 2009, 2011). Given that the recent genome duplication event in maize followed the divergence of maize and sorghum from their common ancestor, we expected that most of the sorghum BAC clones could be used as FISH probes for mapping of two unlinked regions of the maize genome. For example, a sorghum BAC selected with a marker for a locus on maize chromosome 1L may be expected to hybridize as a FISH probe to the corresponding duplicate region on chromosome 5S (Hulbert et al. 1990; Gaut et al. 2000; Lai et al. 2004; Bowers et al. 2005; Ma et al. 2005; Schnable et al. 2009). Our successful test of this prediction with sorghum BACs from locus-specific mapping on maize chromosome 9 highlights the utility of sorghum BACs as robust reagents for detection of maize loci, despite the extensive violations of microcolinearity that characterize different inbred lines of maize (Springer et al. 2009; Woodhouse et al. 2010; Schnable et al. 2011; Schnable and Freeling 2011). A possible limitation of this approach is the use of BACs from regions that are duplicated within sorghum. A single such sorghum BAC might detect up to four loci in maize, although we have not tested this idea directly.

Cytological maps are inherently informative in that they represent direct inspection of loci on maize chromosomes during meiosis. In addition to valuable mapping data, our study can also shed light on the functional organization of the genome at the molecular level. In particular, the mechanism by which long linear DNA molecules are packaged into recombinationally active tubular fibers at meiosis remains enigmatic (Zickler and Kleckner 1998). Even during pachytene, these long fibers undergo axial contraction, but the nature of this process is not well understood. We found that the relative arm positions of loci do not change as a function of chromosome length, so axial contraction at midprophase is relatively uniform, whatever its underlying mechanism.

Our comparative map analyses, using standardized map units, showed that the KYS RN map predictions were in the best overall agreement for marker spacing across the 11 chromosome-arm regions analyzed (Figs. 4 and 5). The relative arm locations vary considerably when linkage maps are compared with physical maps. For example, pericentromeric heterochromatin regions are large, but they typically house few genes and exhibit relatively low recombination rates (Baucom et al. 2009; Liu et al. 2009; Schnable et al. 2009; Talbert and Henikoff 2010). Consequently, genes near centromeres map close together on linkage maps but relatively far apart on physical maps. Our comparisons demonstrated that the relative locus positions on the linkage maps are indeed closer to the centromere than on the cytogenetic maps.

Among the surprising observations resulting from our map comparisons was the variation in marker locations when the cytological and genomic physical maps were compared. We had expected these two maps, both physically based, and with the pachytene fiber appearing relatively uniform in width from end to end, would produce reasonably well-aligned marker placements. On the contrary, we observed considerable variation from one arm to another (Fig. 5c), but within a given arm, the marker offsets were typically skewed in the same direction, diagrammed as individual pairwise alignments in Fig. S3. In some cases the FISH map and the genomic physical map were from different genotypes (Fig. S3, chromosomes 3^Seneca60^ and 6 ^Mo17^), so we were not surprised to see cytological-genomic discrepancies for these, given the vast variation in genomic content typical of inbred lines of *Z. mays*. The genome sizes of the Mo17 and B73 lines are estimated to differ by 0.13 pg, as shown in Table S4. The difference may account for some of the variation observed between the chromosome 6L genomic and cytogenetic RMPs. This idea is supported by findings from Springer et al. (2009), using comparative genomic hybridization, which demonstrated the absence of maize inbred-line B73 megabase-size regions from Mo17. On the other hand, the amounts of DNA in chromosome 3 from B73 and Seneca 60 differ by only 0.06 pg (Table S4). In this case, the variation in RMPs may reflect genotype-specific variation in DNA packaging along the pachytene chromosome axis of individual chromosome arms. Alternatively, the genome sizes may be similar, but repetitive sequences may have accumulated in different regions of the chromosome arms. In fact, the vast intraspecific variations in maize genome organization between inbred lines have been demonstrated extensively at the cytogenetic and molecular levels, as was elegantly reviewed by Llaca et al. (2011). The current genome assembly accounts for 2.07–2.17 Gbp, but estimates place the maize B73 genome size at 2.30–2.55 Gbp (Table S3; http://maizegdb.org/sequencing_project.php; http://www.maizesequence.org/Zea_mays/Info/StatsTable?db=core). These estimates leave several hundred Mbp of genome unaccounted for. The RMPs might therefore appear closer or farther away than they actually are. For example, large segments of mitochondrial organellar genomic DNA that have been inserted into the nuclear genome are not included in the B73 genome assembly. Even at the level of FISH, mitochondrial genomic DNA clearly contributes significantly and differentially to the nuclear genomes of the maize inbred lines (Lough et al. 2008). At least 290 Kb of DNA is estimated to be missing from the Maize B73 genomic sequence as a result of the exclusion of this organellar genomic DNA (Clifton et al. 2004). Some of the local variation across the comparisons including to the B73 genomic RMPs may derive from this incomplete information, confounding to some extent our ability to draw clear conclusions about differential packaging.

Overall, our data do not permit specific elucidation of the basis for the differences we see in marker distribution between cytological and genomic/bp maps (Fig. 5c; Fig. S2), but at least five possible sources for the observed discrepancies can be considered: physical map assembly errors, physical packaging of meiotic chromosome fiber, cytogenetic FISH mapping errors, DNA content differences between the different maize lines, and genetic background effects from the addition lines of oat.

In summary, we report the largest low-copy pachytene FISH mapping study to date in maize, with 29 new loci FISH-mapped with low-copy-number sorghum BAC probes selected with maize RFLP markers for six of the ten chromosomes of maize. Furthermore, we show that sorghum BACs reliably hybridize to two unlinked loci in maize, despite synteny density or even selective marker retention. Collectively, the results extend our knowledge of the maize genome structure at the cytological level while identifying valuable reagents for detection of dispersed low-copy loci in maize.

## Electronic supplementary material

Below is the link to the electronic supplementary material.

Fig. S1 Converting genomic distance to relative genomic map position (RGMP) for two loci. Determining the RGMP of two chromosome 1 loci on the short and long (**c**) Arms. The genomic physical map contigs are annotated with the total chromosome length (Mb), centromere location, length of each arm, as well as the location and genomic distance of a locus on the short (csu3) and long (umc128a) arms (**a**). The calculations used to determine the RGMP of the short- and long-arm loci are also shown in (**b**) and (**c**), respectively. (PPT 210 kb)

Fig. S2 **a** Maize chromosome-9 alignment to regions duplicated on chromosomes 1 and 6. **b** and **c** Physical-chromosome views of the indicated areas in (**a**). The alignments were produced with Symap (Soderlund et al. 2006) annotations of maize contigs as well as an overlay of known (*solid lines*) or approximate (*dashed lines*) maize bin locations annotated with the Maize B73 RefGen v2 (Schnable et al. 2009; Sen et al. 2009; http://gbrowse.maizegdb.org/cgi-bin/gbrowse/maize_v2/). (PPT 674 kb)

Fig. S3 Comparison of the cytogenetic pachytene FISH karyotypes to the relative map position on the genomic physical map. The chromosome number and maize inbred line source are indicated in superscript, and all the chromosomes are aligned at the centromere with the genomic physical karyotype displayed above the cytogenetic FISH karyotype. Chromosome arm ratios are drawn to scale as per Anderson et al. (2004) with the same color scheme used in Fig. 5. (PPT 627 kb)

Supplemental Table 1 Chromosome arm ratios from various studies (DOC 35 kb)

Supplemental Table 2 Maize duplicate regions 9–6 and 9–1 (DOC 66 kb)

Supplemental Table 3 The relative genomic physical map positions (RMPs) of FISH-mapped loci (DOC 66 kb)

Supplemental Table 4 Genome-size (*C* value) estimates for maize (*Z. mays* ssp. *mays*) inbred lines (DOC 34 kb)

## Abbreviations

BAC: Bacterial artificial chromosome
cM: CentiMorgan
cMC: CentiMcClintock
FISH: Fluorescence in situ hybridization
RFLP: Restriction fragment length polymorphism
RMP: Relative map position
RN: Recombination nodule
