# Supplementary material for: Development of pachytene FISH maps for six maize chromosomes and their integration with other maize maps for insights into genome structure variation
Source: Chromosome Res. 2012 May 16;20(4):363–80. doi: 10.1007/s10577-012-9281-4 (PMC3391363; doi:10.1007/s10577-012-9281-4)
Supplement: Supplementary file 1 — Converting genomic distance to relative genomic map position (RGMP) for two loci. Determining the RGMP of two chromosome 1 loci on the short and long (c) Arms. The genomic physical map contigs are annotated with the total chromosome length (Mb), centromere location, length of each arm, as well as the location and genomic distance of a locus on the short (csu3) and long (umc128a) arms (a). The calculations used to determine the RGMP of the short- and long-arm loci are also shown in (b) and (c), respectively. (PPT 210 kb) [file 10577_2012_9281_MOESM1_ESM.ppt]

## Slide 1
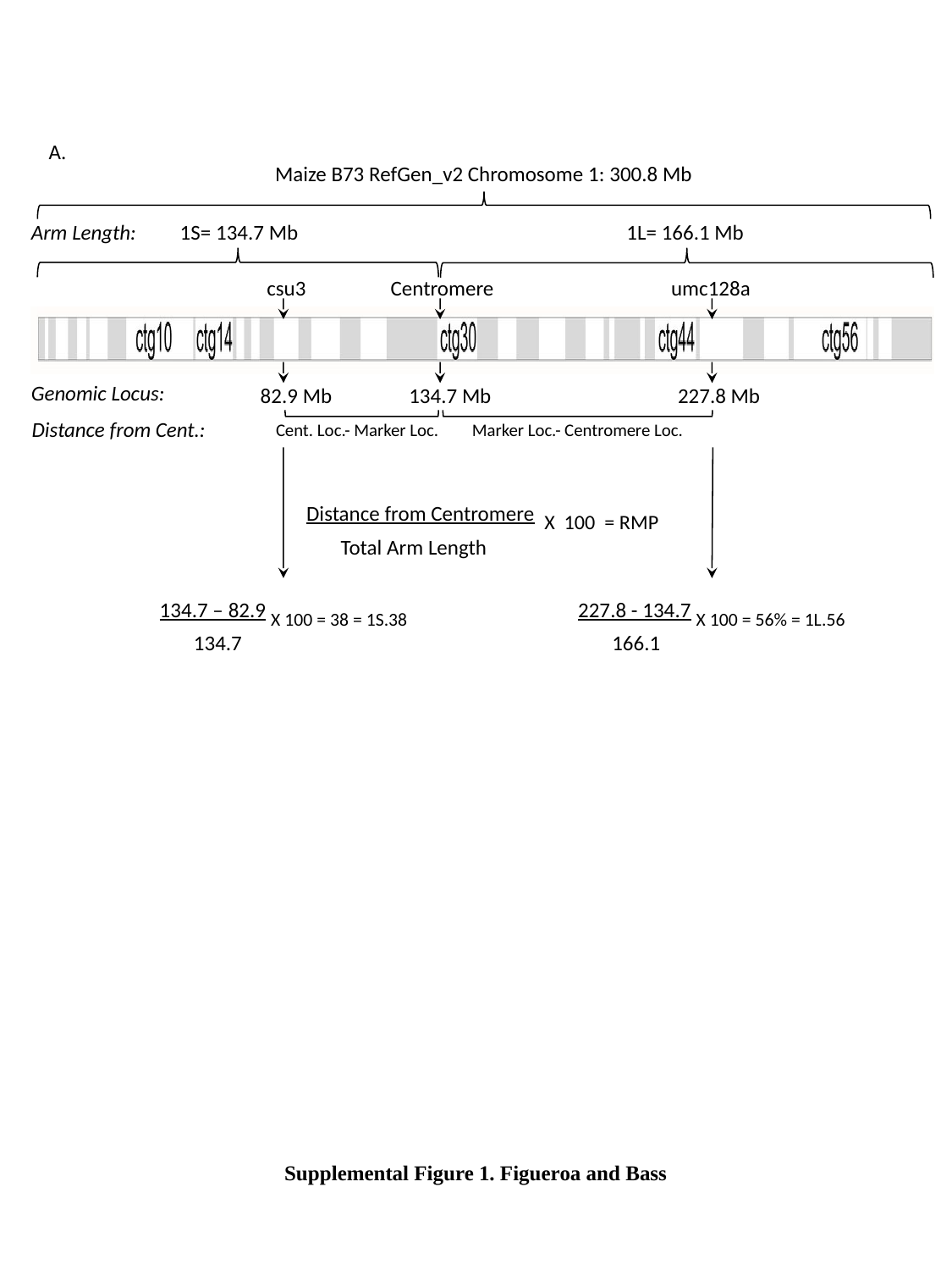

A.
Maize B73 RefGen_v2 Chromosome 1: 300.8 Mb
Arm Length:
1S= 134.7 Mb
1L= 166.1 Mb
csu3
Centromere
umc128a
Genomic Locus:
82.9 Mb
134.7 Mb
227.8 Mb
Distance from Cent.:
Cent. Loc.- Marker Loc.
Marker Loc.- Centromere Loc.
Distance from Centromere X 100 = RMP
 Total Arm Length
134.7 – 82.9 X 100 = 38 = 1S.38
 134.7
227.8 - 134.7 X 100 = 56% = 1L.56
 166.1
Supplemental Figure 1. Figueroa and Bass
