# Supplementary material for: Development of pachytene FISH maps for six maize chromosomes and their integration with other maize maps for insights into genome structure variation
Source: Chromosome Res. 2012 May 16;20(4):363–80. doi: 10.1007/s10577-012-9281-4 (PMC3391363; doi:10.1007/s10577-012-9281-4)
Supplement: Supplementary file 2 — a Maize chromosome-9 alignment to regions duplicated on chromosomes 1 and 6. b and c Physical-chromosome views of the indicated areas in (a). The alignments were produced with Symap (Soderlund et al. 2006) annotations of maize contigs as well as an overlay of known (solid lines) or approximate (dashed lines) maize bin locations annotated with the Maize B73 RefGen v2 (Schnable et al. 2009; Sen et al. 2009; http://gbrowse.maizegdb.org/cgi-bin/gbrowse/maize_v2/). (PPT 674 kb) [file 10577_2012_9281_MOESM2_ESM.ppt]

## Slide 1
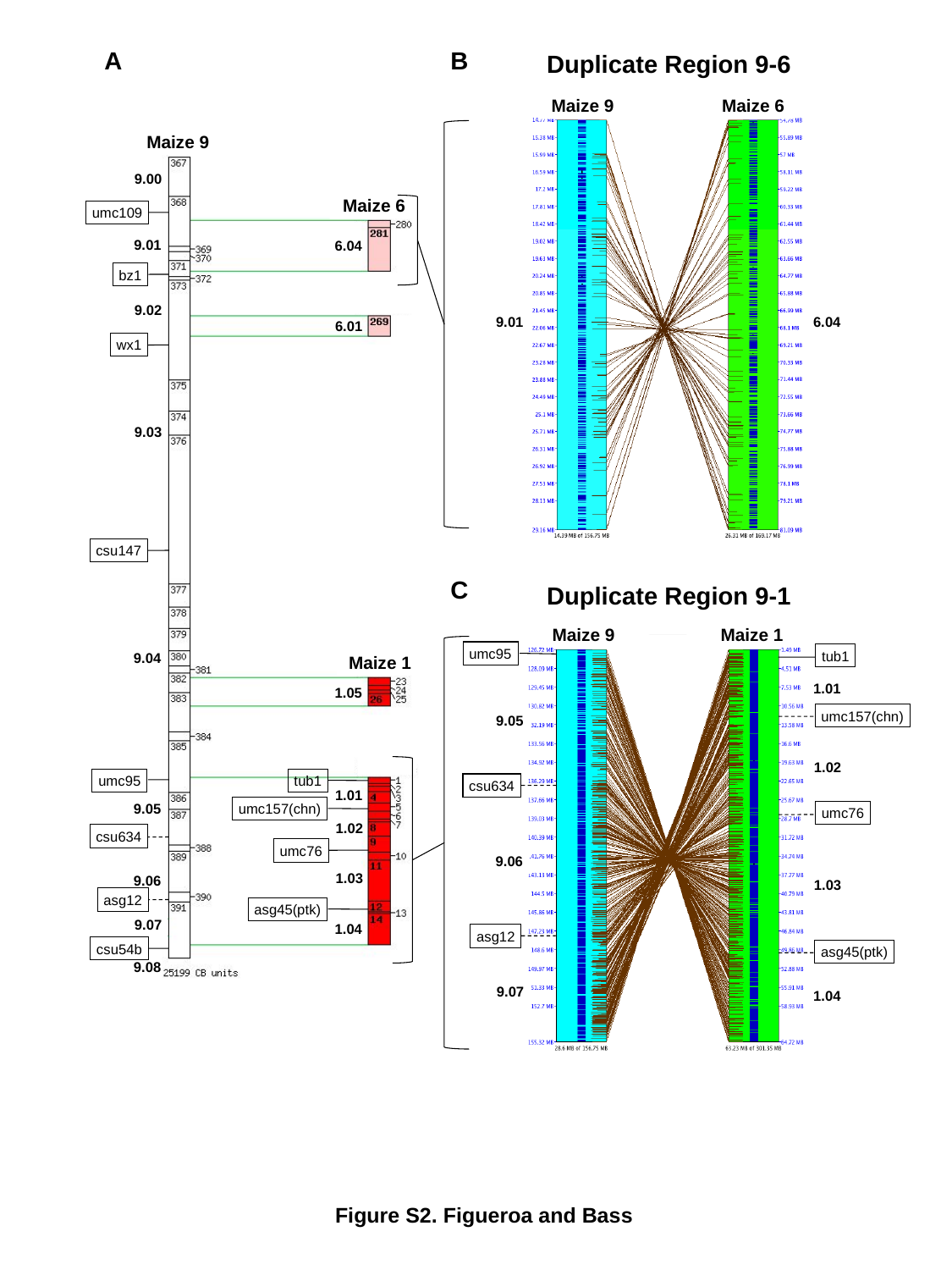

Duplicate Region 9-6
A
B
Maize 9
Maize 6
Maize 9
9.00
Maize 6
umc109
9.01
6.04
bz1
9.02
9.01
6.04
6.01
wx1
9.03
csu147
C
Duplicate Region 9-1
Maize 9
Maize 1
umc95
tub1
9.04
Maize 1
1.01
1.05
umc157(chn)
9.05
1.02
umc95
tub1
csu634
1.01
9.05
umc157(chn)
umc76
1.02
csu634
umc76
9.06
1.03
9.06
1.03
asg12
asg45(ptk)
9.07
1.04
asg12
csu54b
asg45(ptk)
9.08
9.07
1.04
Figure S2. Figueroa and Bass
