# Supplementary material for: Development of pachytene FISH maps for six maize chromosomes and their integration with other maize maps for insights into genome structure variation
Source: Chromosome Res. 2012 May 16;20(4):363–80. doi: 10.1007/s10577-012-9281-4 (PMC3391363; doi:10.1007/s10577-012-9281-4)
Supplement: Supplementary file 3 — Comparison of the cytogenetic pachytene FISH karyotypes to the relative map position on the genomic physical map. The chromosome number and maize inbred line source are indicated in superscript, and all the chromosomes are aligned at the centromere with the genomic physical karyotype displayed above the cytogenetic FISH karyotype. Chromosome arm ratios are drawn to scale as per Anderson et al. (2004) with the same color scheme used in Fig. 5. (PPT 627 kb) [file 10577_2012_9281_MOESM3_ESM.ppt]

## Slide 1
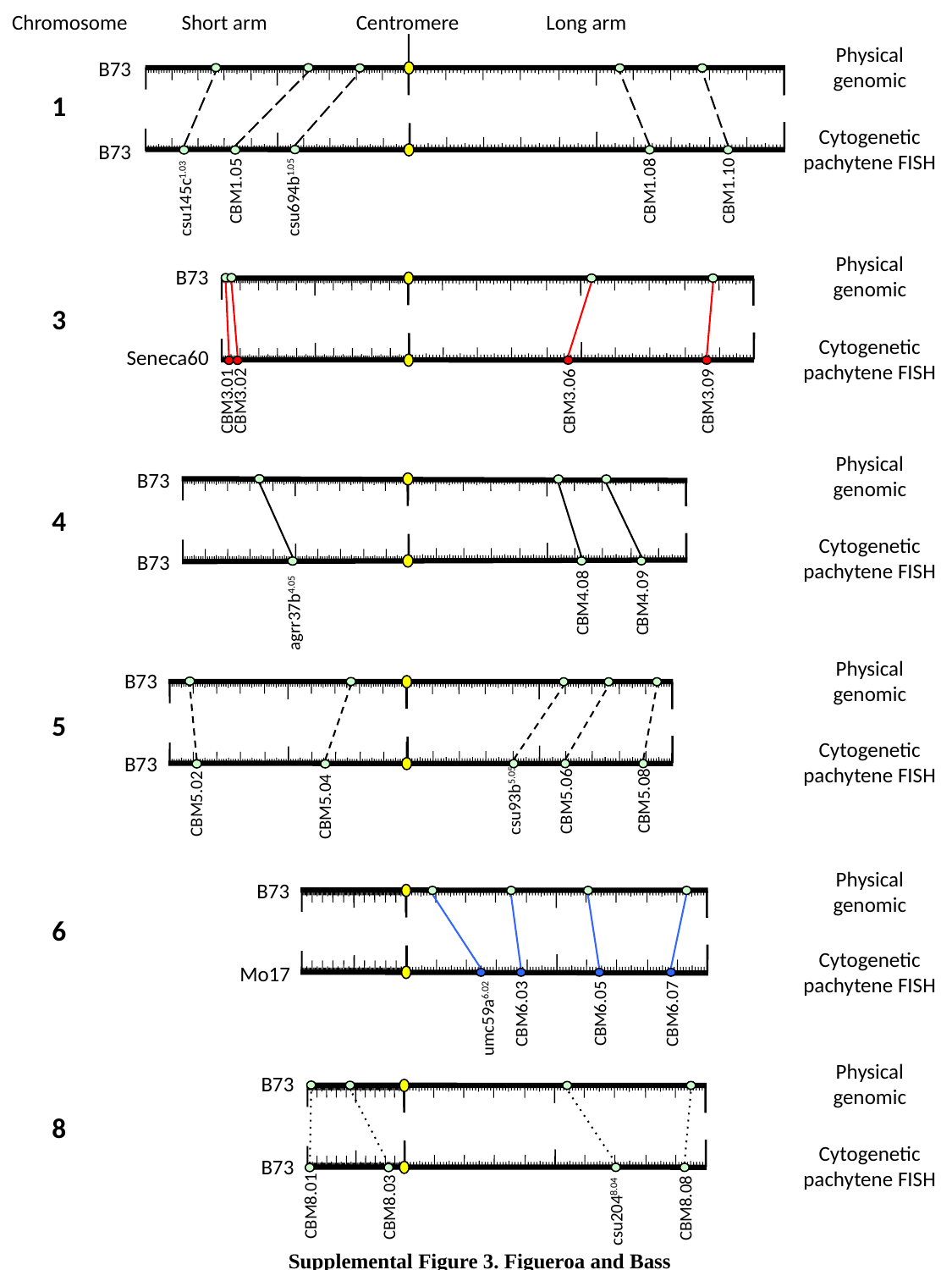

Chromosome
Short arm
Centromere
Long arm
Physical genomic
B73
1
Cytogenetic pachytene FISH
B73
CBM1.05
CBM1.08
CBM1.10
csu694b1.05
csu145c1.03
Physical genomic
B73
3
Cytogenetic pachytene FISH
Seneca60
CBM3.02
CBM3.01
CBM3.09
CBM3.06
Physical genomic
B73
4
Cytogenetic pachytene FISH
B73
CBM4.08
CBM4.09
agrr37b4.05
Physical genomic
B73
5
Cytogenetic pachytene FISH
B73
CBM5.08
CBM5.02
CBM5.04
csu93b5.05
CBM5.06
Physical genomic
B73
6
Cytogenetic pachytene FISH
Mo17
CBM6.05
CBM6.03
CBM6.07
umc59a6.02
Physical genomic
B73
8
Cytogenetic pachytene FISH
B73
CBM8.01
CBM8.03
CBM8.08
csu2048.04
Supplemental Figure 3. Figueroa and Bass
