# Supplementary material for: Development of pachytene FISH maps for six maize chromosomes and their integration with other maize maps for insights into genome structure variation
Source: Chromosome Res. 2012 May 16;20(4):363–80. doi: 10.1007/s10577-012-9281-4 (PMC3391363; doi:10.1007/s10577-012-9281-4)
Supplement: Supplementary file 4 — Chromosome arm ratios from various studies (DOC 35 kb) [file 10577_2012_9281_MOESM4_ESM.doc]

Supplemental Table 1. Chromosome arm ratios from various studies.

| Chromo-some | Rhoades^a^ (1950) | Bennett and Laurie^b^ (1995) | Neuffer et al.^a,c^ (1997) | Chen et al.^a^ (2000) | Sadder & Weber^d^ (2001) | Wang et al.^a,e^ (2006) | Present study^f^ |
| --- | --- | --- | --- | --- | --- | --- | --- |
| 1 | 1.30 | 1.20 | 1.23 | 1.31 ± 0.13 | 1 (1:1) | 1.30 | 1.47 ± 0.02 (210)^B73^ |
| 3 | 2.00 | 1.84 | 2.00 | 2.07 ± 0.13 | 2 (1:2) | 2.02 | 1.84 ± 0.03 (56)^Seneca 60^ |
| 4 | 1.60 | 1.59 | 1.63 | 1.67 ± 0.18 | 2 (1:2) | 1.58 | 1.24 ± 0.02 (44)^B73^ |
| 5 | 1.10 | 1.10 | 1.07 | 1.06 ± 0.09 | 1 (1:1) | 1.07 | 1.11 ± 0.01 (79)^B73^ |
| 6 | 7.10, 3.10^g^ | 1.92 | 3.10 | 4.33 ± 0.71 | 2 (1:2) | 3.49 | 2.83 ± 0.06 (111)^Mo17^ |
| 8 | 3.20 | 2.60 | 3.00 | 3.42 ± 0.44 | 3 (1:3) | 3.16 | 3.01 ± 0.06 (73)^B73^ |

^a^Cell type = meiotic (pachytene); genotype = KYS.

^b^Cell type = meiotic (pachytene); genotype = Seneca60.

^c^A consensus of various studies.

^d^Cell type = somatic (metaphase); genotype = KYS.

^e^Values obtained by squash technique.

^f^Cell type = meiotic (pachytene); genotype used for each chromosome is indicated by superscript.

^g^Dempsey et al (1994)
