# Supplementary material for: Development of pachytene FISH maps for six maize chromosomes and their integration with other maize maps for insights into genome structure variation
Source: Chromosome Res. 2012 May 16;20(4):363–80. doi: 10.1007/s10577-012-9281-4 (PMC3391363; doi:10.1007/s10577-012-9281-4)
Supplement: Supplementary file 5 — Maize duplicate regions 9–6 and 9–1 (DOC 66 kb) [file 10577_2012_9281_MOESM5_ESM.doc]

Supplemental Table 2. Maize duplicate regions 9-6 and 9-1.

|  | Chromosome 9 coordinates | | | Duplicate region | |
| --- | --- | --- | --- | --- | --- |
| Maize RFLP | Selected homologous sorghum BAC^a^ | Cytogenetic locus name^a^ | Genomic coordinates^b^ (Mb) | Duplicate locus name: bin^c^ | Genomic coordinates^b^ (Mb) |
| Duplicate Region 9-6 | | | | | |
| rz144a | a0030K10 | spb-9.01_S82 | 3.6 (2.9–4.3)^d^ | rz144b: 6.04 | 117.0 (116.1–118.0) |
| umc109 | a0004M18 | spb-CBM9.01_S79 | 2.5 (2.2–2.9)^d^ | (no mapped duplicate) |  |
| rz144c | a0030K10 | spb-9.01_S74 | 6.1 (4.4–7.7)^d^ | (no mapped duplicate) |  |
| php10005 | a0045A20 | spb-9.01_S73 | 5.66^e^ | (no mapped duplicate) |  |
| csu95a | a0012H11 | spb-9.01_S67 | 7.6 (7.4–7.7)^d^ | csu95d: 6.04g |  |
| sh1 | a0015M03 | spb-9.01_S66 | 11.50^e^ | (no mapped duplicate) |  |
| bz1 | a0020G06 | spb-CBM9.02_S65 | 11.78^e^ | (no mapped duplicate) |  |
| Duplicate Region 9-1 | | | | | |
| Xtxa325g | sbb18256 | sbb-9.04_L27 |  | (no mapped duplicate) |  |
| csu694a (uce) | a0093O18 | spb-9.04_L37 | 127.7 (127.5–127.9)^d^ | csu694b(uce): 1.05 | 109.8 (84.8–221.3)^h^ |
| umc95 | a0063J06 | spb-CBM9.05_L38 | 126.93^e^ | (no mapped duplicate) |  |
| csu392a | a0010M15 | spb-9.05_L42 | 132.6 (132.0–133.2)d | csu392b: 1.03 | 51.6 (51.3–52.0) |
| csu710e (apx) | a0019J05 | spb-9.05_L48 | 132.6 (130.9–134.3)^d^ | csu710a: 1.03 | 43.3 (43.1–43.5) |
|  |  |  |  | csu710f: 1.05 | 165.4 |
| csu219 (tgd) | a0059O14 | spb-9.05_L50 | 132.6 (130.9–134.3)^d^ | (no mapped duplicate) |  |
| csu59a | a0074G20 | spb-9.06_L53 | 132.6 (130.9–134.3)^d^ | csu59b: 1.03g |  |
| csu145a (pck) | a0055A21/a0093D20 | spb-9.06_L53 | 142.4 (142.4–142.5)^d^ | csu145c(pck): 1.02 | 35.4 (34.9–35.8) |
| Xtxp32g | sbb16685 | spb-9.06_L53 |  | (no mapped duplicate) |  |
| csu28a (rpS22) | a0093D21 | spb-9.06_L54 | 142.8 (142.2–143.4)^d^ | (no mapped duplicate) |  |
| asg44 | a0033A22 | spb-9.06_L63 | 142.8 (142.2–143.4)^d^ | (no mapped duplicate) |  |
| cdo1387a (emp70) | a0036J08/a0036F07 | spb-9.06_L73 | 144.8 (144.6–145.0)^d^ | cdo1387b(emp70): 1.03 | 27.7 (27.5–27.9) |
| csu1004 | a0046P22 | spb-9.06_L77 | 147.1 (146.9–147.4)^d^ | (no mapped duplicate) |  |
| asg12a | a0064E21 | spb-CBM9.07_L78 | 148.3 (147.9–148.8)^d^ | (no mapped duplicate) |  |
| csu1118 | a0078D03 | spb-9.07_L83 | 149.7 (149.5–149.8)^d^ | (no mapped duplicate) |  |
| csu285 (his2B) | a0026P15 | spb-9.07_L94 | 154.7 (154.4–155.1)^d^ | (no mapped duplicate) |  |
| csu54b | a0074A03 | spb-CBM9.08_L95 | 154.1 (152.4–156.0)^d^ | (no mapped duplicate) |  |

^a^As previously published by Amarillo and Bass (2007).

^b^Physical coordinates as obtained from maize B73 RefGen_v2.

^c^Alternate locus names and corresponding bin denoted only for loci in the duplicated syntenic blocks under investigation.

^d^Absolute sequence location estimated from mapped closest flanking loci.

^e^Locus location from known physical coordinates on Maize RefGen_v2.

^g^Marker coordinates are not annotated on Maize RefGen_v2.

^h^Estimated coordinates (from maizegdb.org) used a large range that spanned both long and short arms. We therefore used the mean distance from centromere to the most distal value on the appropriate arm: 1.05 [csu694(uce)]: 135.5 Mb range (TIDP6240-TIDP6245).
