# Supplementary material for: Development of pachytene FISH maps for six maize chromosomes and their integration with other maize maps for insights into genome structure variation
Source: Chromosome Res. 2012 May 16;20(4):363–80. doi: 10.1007/s10577-012-9281-4 (PMC3391363; doi:10.1007/s10577-012-9281-4)
Supplement: Supplementary file 6 — The relative genomic physical map positions (RMPs) of FISH-mapped loci (DOC 66 kb) [file 10577_2012_9281_MOESM6_ESM.doc]

Supplemental Table 3. The relative genomic physical map positions (RMPs) of FISH-mapped loci

|  |  |  | Conversion of genomic coordinates to fractional distance on chromosome arm^a^ | | | | | |
| --- | --- | --- | --- | --- | --- | --- | --- | --- |
| bin | Locus | Arm^b^ | Centromere location^c^ | Total chromosome length (Mb) | Total arm length^d^ (Mb) | Location on physical chromosome (Mb) | Distance from centromere^e^ (Mb) | Relative map position^f^ % (RMP range) |
| CBM1.05 | csu3^g^ | S | 134.7 | 300.8 | 134.7 | 82.6 | 52.1 | 39S |
| CBM1.08 | umc128^ah^ | L |  |  | 166.1 | 227.8 (227.0–228.6) | 93.1 | 56L (56L–57L) |
| CBM1.10 | umc107a(croc)^h^ | L |  |  | 166.1 | 264.7 (263.2–266.2) | 130.0 | 78L (77L–79L) |
| CBM3.01 | umc032a^h^ | S | 100.2 | 232.1 | 100.2 | 2.1 (2.1–2.1) | 98.1 | 98S |
| CBM3.02 | csu32a^h^ | S |  |  | 100.2 | 4.1 (3.8–4.4) | 96.1 | 96S |
| CBM3.06 | bnl5.37a^h^ | L |  |  | 131.9 | 169.5 (167.4–169.6) | 69.3 | 53L |
| CBM3.09 | umc63a^h^ | L |  |  | 131.9 | 215.9 (215.6–216.1) | 115.7 | 88L (87L–88L) |
| 4.05 | agrr37b^h^ | S | 105.7 | 241.7 | 105.7 | 36.3 (32.1–40.4) | 69.4 | 66S (62S–69S) |
| CBM4.08 | umc127c^h^ | L |  |  | 136.0 | 179.7 (179.6–179.8) | 74.0 | 54L |
| CBM4.09 | umc52a^h^ | L |  |  | 136.0 | 202.2 (199.8–204.6) | 96.5 | 71L (69L–73L) |
| CBM5.02 | umc90^h^ | S | 105.8 | 217.9 | 105.8 | 8.5 (7.6–9.5) | 97.2 | 92S (91S–93S) |
| CBM5.04 | bnl4.36^g^ | S |  |  | 105.8 | 80.80 | 24.9 | 24S |
| 5.05 | csu93b^h^ | L |  |  | 112.1 | 171.5 (171.0–172.0) | 65.7 | 59L (58L–59L) |
| CBM5.06 | umc126^h^ | L |  |  | 112.1 | 190.9 (190.7–191.1) | 85.2 | 76L |
| CBM5.08 | bnl524a^h^ | L |  |  | 112.1 | 211.2 (205.6–216.9) | 105.5 | 94L (89L–99L) |
| 6.02 | umc59a^i^ | L | 49.8 | 169.2 | 119.4 | 60.4 (49.8–71.0) | 10.6 | 9L (0L–18L) |
| CBM6.03 | npi393^h^ | L |  |  | 119.4 | 91.6 (91.2–92.1) | 41.8 | 35L |
| CBM6.05 | umc21^g^ | L |  |  | 119.4 | 120.90 | 71.1 | 60L |
| CBM6.07 | umc132a(chk)^h^ | L |  |  | 119.4 | 161.3 (161.1–161.6) | 111.5 | 93L (93L–94L) |
| CBM8.01 | npi220a^g^ | S | 50.2 | 175.8 | 50.2 | 1.8 | 48.4 | 96S |
| CBM8.03 | umc124a(chk)^h^ | S |  |  | 50.2 | 22.1 (21.9–22.3) | 28.2 | 56S |
| 8.04 | csu204(uce)^h^ | L |  |  | 125.6 | 118.6 (118.1–119.0) | 68.4 | 54L (54L–55L) |
| CBM8.08 | npi414a^g^ | L |  |  | 125.6 | 169.9 | 119.7 | 95L |
| Duplicate bin | | | | | | | | |
| 6.04 | csu95d^j^ | L | 49.8 | 169.2 | 49.8 | — |  |  |
| 1.05 | csu694b(uce)^i^ | S | 134.7 | 300.8 | 134.7 | 109.4 (84.8–134.7) | 49.9 | 19S (0S–37S) |
| 1.03 | csu59b^j^ | S |  |  | 134.7 | — |  |  |
| 1.02–1.03 | csu145c^h^ | S |  |  | 134.7 | 35.4 (34.9–35.8) | 99.4 | 74S (73S–74S) |

^a^Based on Maize B73 RefGen_v2.

^b^Chromosome arm on which locus resides, long (L) or short (S).

^c^Wolfgruber et al. (2009) centromere position annotations updated from Maize RefGen_v2.

^d^Short arm length = Centromere location; Long arm length = Total chromosome length – Centromere location.

^e^Distance from centromere: Short arm = Centromere location – Locus location; Long arm = Locus location – Centromere location.

^f^Relative map position (RMP) = (Distance from centromere/Total arm length) × 100. The rage of RMP values is provided when the provided flanking loci are at least 1 RMP unit apart.

^g^Locus sequence location with actual physical coordinates.

^h^Absolute sequence location based on estimate using closest flanking mapped loci.

^i^MaizeGDB–estimated coordinates used a large range that spanned both long and short arms. We therefore used the mean distance from centromere to the most distal value on the appropriate arm: 6.02 (umc59a): 33 Mb range (IDP680-phi007); 1.05 (csu694b): 127 Mb range (TIDP6240-TIDP6245).

^k^MaizeGDB was "unable to find coordinates for csu59b on the B73 RefGen_v2 sequence."
