# Supplementary material for: Development of pachytene FISH maps for six maize chromosomes and their integration with other maize maps for insights into genome structure variation
Source: Chromosome Res. 2012 May 16;20(4):363–80. doi: 10.1007/s10577-012-9281-4 (PMC3391363; doi:10.1007/s10577-012-9281-4)
Supplement: Supplementary file 7 — Genome-size (C value) estimates for maize (Z. mays ssp. mays) inbred lines (DOC 34 kb) [file 10577_2012_9281_MOESM7_ESM.doc]

Supplemental Table 4. Genome-size (C-value) estimates for maize (*Zea mays* ssp. *mays*) inbred lines.

| Maize inbred line | C-value (pg) | Genome size (Gbp)^a^ | Reference |
| --- | --- | --- | --- |
| Mo17 | 2.38 | 2.33 | Rayburn et al. 1993 |
| B73 | 2.43–2.58 | 2.38–2.52 | Rayburn et al. 1985; Lee et al. 2002 |
| Seneca 60 | 2.46 | 2.41 | Laurie and Bennett 1985; Bennett and Laurie 1995 |
| KYS | 2.76–2.8 | 2.70–2.74 | Laurie and Bennett 1985; Rayburn et al. 1985; Lee et al. 2002 |

^a^Conversion factor of 1 pg = 0.978 Gbp as described by Doležel et al. (2003).
